# Value of ultrasound in the anatomical evaluation of the brachial plexus: correlation with magnetic resonance imaging

**DOI:** 10.1590/0100-3984.2017.0083

**Published:** 2018

**Authors:** Wanda Chiyoko Iwakami Caldana, Sergio Keidi Kodaira, Conrado Furtado de Albuquerque Cavalcanti, Marcelo Bordalo Rodrigues, Osmar de Cassio Saito, Carlos Alberto Buchpiguel

**Affiliations:** 1 Instituto de Radiologia do Hospital das Clínicas da Faculdade de Medicina da Universidade de São Paulo (InRad/HC-FMUSP), São Paulo, SP, Brazil.

**Keywords:** Brachial plexus/anatomy & histology, Ultrasonography, Magnetic resonance imaging

## Abstract

**Objective:**

To assess the accuracy of ultrasound in the visualization of the brachial plexus and to determine the value of the method in comparison with that of magnetic resonance imaging (MRI).

**Materials and Methods:**

This was an anatomical study of the brachial plexuses of 20 asymptomatic adults (40 plexuses), comparing ultrasound and MRI in terms of their accuracy. In the ultrasound study, a high-frequency linear transducer was used, and a neurovascular coil was used in the MRI study. To estimate the frequency of visualization, the brachial plexus was divided into segments.

**Results:**

The cervical nerve roots, the upper trunk, and the middle trunk were the segments that were best visualized on ultrasound. On MRI, the degree of visualization was excellent for most of the segments. In the comparison between ultrasound and MRI, the C6, C7, upper trunk, and middle trunk segments showed equivalent degrees of visualization, with a high level of agreement between the two methods.

**Conclusion:**

In the brachial plexus, ultrasound can be used in the assessment of the cervical nerve roots, as well as of the upper and middle trunks, although it provides limited visualization of the remaining segments. Ultrasound and MRI showed a high level of agreement for the visualization of the C6, C7, and middle trunk segments.

## INTRODUCTION

The brachial plexus is a network of nerve structures responsible for motor and sensory innervation of the upper limb. It is composed of the ventral branches of the C5-T1 nerve roots, which originate in the posterolateral region of the neck and pass through the intervertebral foramina; as they pass through the lateral cervicothoracic region (which comprises the interscalene triangle, costoclavicular space, and retropectoralis minor space), they unite or split to form the trunks, divisions, and cords of the plexus^(^^1^^)^. The peripheral nerves of the upper limbs originate from the division of the cords. The brachial plexus is located in the same region as the thoracic and axillary structures, which include the subclavian vessels, as well as the musculature and bony framework and of the cervical and thoracic regions, together with fat and the pulmonary apex, constituting a complex internal anatomy, which makes it a challenge to perform appropriate imaging studies of this region^(^^2^^)^.

The main conditions that affect the structures of the thoracic gorge, resulting in neuromotor and vascular disorders, are as follows: traumatic plexopathy (caused by dystocia in neonates and by motorcycle accidents in adults); compressive plexopathy along the neurovascular pathway, caused by tumors; anatomical variations or fibrotic bands; and plexopathies caused by tumor infiltration or radiotherapy. Imaging methods are of fundamental importance as complementary diagnostic tools, not only for identifying the location of an injury affecting the brachial plexus but also for defining its characteristics^(^^2^^)^.

Despite its cost, magnetic resonance imaging (MRI) is considered one of the best imaging methods for assessing the brachial plexus. It has the advantages of being a noninvasive method that does not involve the use of ionizing radiation, as well as being able to show anatomical features in greater detail, because of its multiplanar acquisition and the high degree of contrast it creates between different tissue types^(^^3^^-^^5^^)^. However, MRI does have some limitations, such as the considerable time required for image acquisition and the occasional use of paramagnetic contrast (gadolinium), as well as restrictions presented by patients, such as claustrophobia or metal implants, all of which impair the quality and impede the analysis of the images acquired^(^^2^^,^^3^^)^.

Ultrasound of the brachial plexus was first employed as an auxiliary method for procedures involving nerve block anesthesia. Recent studies have demonstrated that ultrasound is sufficiently accurate in identifying the cervical roots that make up the brachial plexus, mainly by allowing visualization of the path the roots take as they merge to form the trunks in the interscalene space. In comparison with MRI, ultrasound has the advantage of being a more rapid method and is equally non-invasive. Unlike MRI, it is widely available, is affordable, and has no contraindications. Ultrasound can be used dynamically, enabling provocative maneuvers that are of fundamental importance in thoracic outlet syndrome, as opposed to MRI, which requires the patient to be in a static position, with limited space for changing the position of the arms. Therefore, ultrasound is an additional imaging method that may contribute to the characterization of changes that affect the brachial plexus, although there is as yet insufficient evidence of its value^(^^6^^-^^9^^)^.

The limited knowledge and underuse of brachial plexus ultrasound prompted our interest in developing this study. Our objective was to assess the effectiveness of ultrasound in plexus visualization, as well as its value in comparison with MRI.

## MATERIALS AND METHODS

This was a comparative study of ultrasound and MRI, studying the anatomy of the right and left brachial plexuses of 20 volunteers. For inclusion in the study, the criteria were being conscious, being collaborative, and being over 18 years of age. We excluded individuals in whom MRI examination was contraindicated, as well as those with a history of alterations or symptoms related to the brachial plexus. The study group was composed of 10 men and 10 women between 33 and 68 years of age (mean age, 47.2 years). The ultrasound study was conducted with a Acuson Antares Premium Edition ultrasound system (Siemens Medical Solutions, Erlangen, Germany) with a linear transducer (5-13 mHz), and the MRI study was conducted with a 1.5 T Signa Excite HDX scanner (GE Healthcare, Chicago, IL, USA) using a neurovascular coil.

The ultrasound examinations of the region extending from the extraforaminal nerve roots up to the interscalene space were executed with the patient lying down, with the neck in a neutral position and slightly bent toward the contralateral side. The visualization of that trajectory and the components of the brachial plexus was approached as described below.

**Figure 1:**
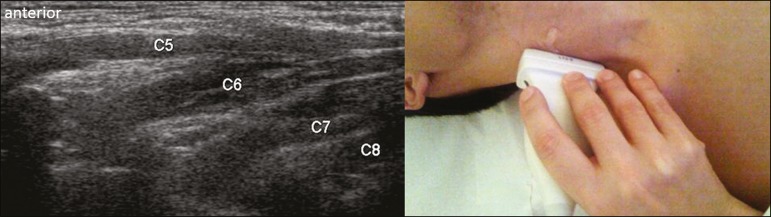
Ultrasound in the longitudinal plane, showing the C5-C8 roots.

**Roots from C5 to C8** - In the anterior region of the neck, we positioned the transducer in the longitudinal direction, parallel to the body axis, with a slight inclination of the upper border to the posterior region (coronal oblique). The visualization of the cervical roots began from C5 to T1, with the identification of the first rib. The passage of the subclavian artery over the rib was used as an anatomical reference. In this region, we were able to visualize the C8 root, the first root that is situated above the rib. From that reference point, we located all the roots that emerge from the intervertebral foramina (the C5, C6, and C7 roots), which were subsequently counted and identified, from the bottom up (Figure 1).

**Figure 2:**
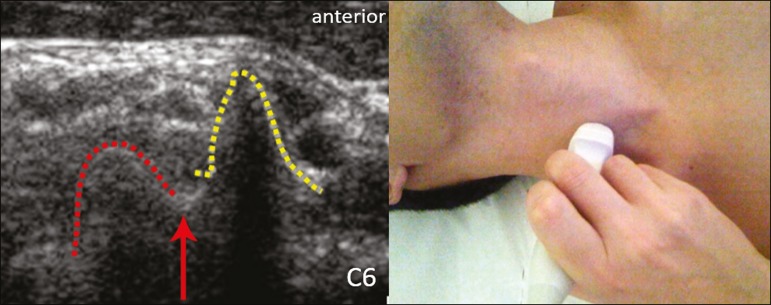
Ultrasound showing the C6 root (arrow), as well as the anterior and posterior tubercles of the transverse process (in yellow and red, respectively).

**Figure 3:**
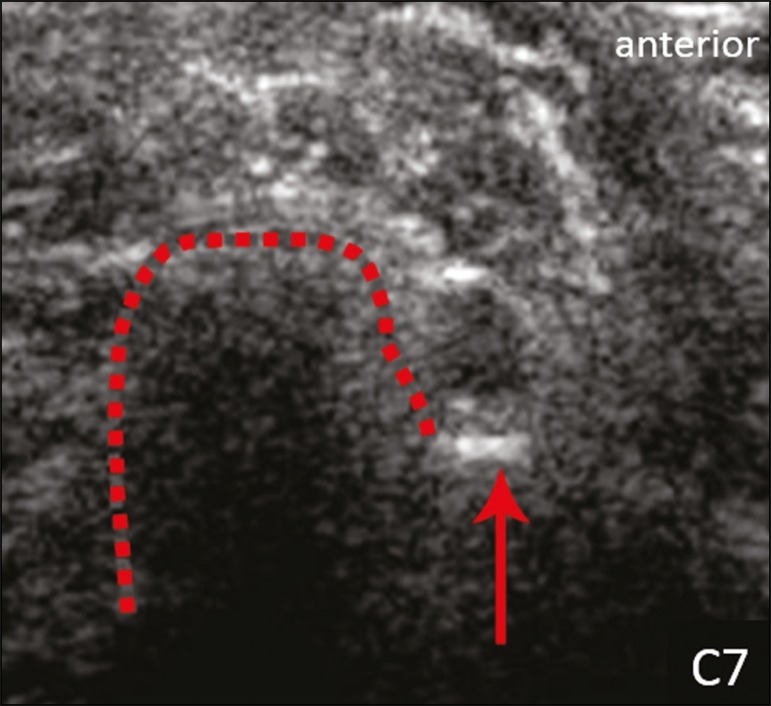
Ultrasound in the transverse plane, showing the C7 root (arrow) and the posterior transverse process (red line).

**Figure 4:**
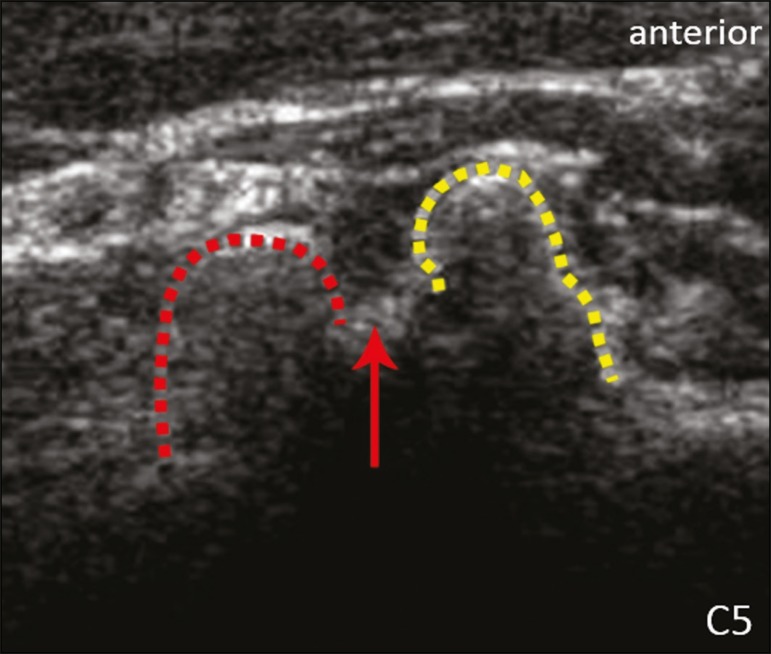
Ultrasound in the transverse plane, showing the C5 root (arrow), anterior tubercles (yellow line) and posterior tubercles (red line).

As a complement to and aid for the identification of roots, we used the morphology of the anterior and posterior tubercle of the transverse processes of the cervical vertebrae. For the characterization of the C6 root, we looked for the anterior and posterior tubercle of the transverse process, which are similar in height and morphology (Figure 2). The C7 root is identified in the transverse process region, where the posterior tubercle is the only prominent feature (Figure 3). After identifying the C6 root, we kept the transducer in the transverse plane and moved it upward until we reached the anterior and posterior tubercle of the C5 transverse process, which are closer than are those of the C6 transverse process (Figure 4).

**Interscalene space** - To identify the upper, middle, and lower trunks, we followed the C7 root from the paravertebral region of the neck to the space between the anterior and middle scalene muscles, where the trunks of the plexus form, with the transducer in the longitudinal plane. After the muscles had been identified, we switched the axis of the transducer to the transverse direction, bringing it perpendicular to the neck, which allowed us to identify the middle trunk. In that same region, the upper trunk will be superior to the middle trunk and the lower trunk, in the inferior region.

**Figure 5:**
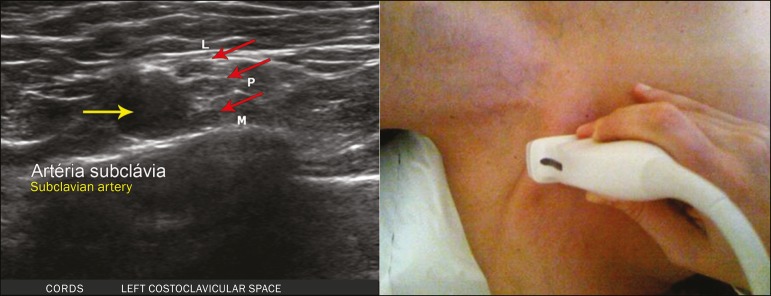
Ultrasound showing the costoclavicular space, with the transducer in the longitudinal plane, together with the lateral (L), medial (M), and posterior (P) cords (red arrows) around the subclavian artery (yellow arrow).

**Transition between the interscalene and costoclavicular spaces** - With the transducer transverse to the shoulder axis, we visualized the divisions of the trunks in the supraclavicular region, our reference being the middle-third clavicle. The divisions of the trunks are above the subclavian artery (Figure 5).

**Costoclavicular infraclavicular space** - With the transducer in the longitudinal direction, parallel to the axis of the body, we identified the subclavian artery, immediately below and lateral to the middle/distal third clavicle. In relation to the subclavian artery, the lateral cord is the most superficial and anterior, the posterior cord being located in the upper portion and the medial cord being located in the posterior portion.

**Figure 6:**
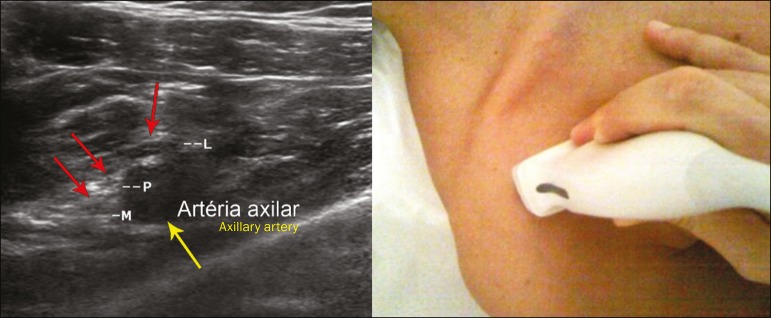
Ultrasound of the retropectoralis minor space with the transducer in the transversal plane showing the medial (M), lateral (L), and posterior (P) cords (yellow arrows).

**Retropectoralis minor space** - With the transducer positioned longitudinally, parallel to the body's axis, we identified the axillary artery, at the level of the coracoid process and at the distal third of the articular clavicle. Of the brachial plexus cords, the lateral cord is the most superficial and anterior to the axillary artery. The posterior cord is located superior to the axillary artery, and the medial cord is located posterior to the axillary artery (Figure 6).

MRI exams were performed in sagittal T1-weighted FSE sequences of the cervical spine and bilateral brachial plexus, from the intervertebral foramen to the edge of the scapula (slice thickness, 4 mm); in oblique coronal T2-weighted sequences of the right and left plexuses (slice thickness, 3 mm); and in a coronal STIR sequence of the right cervicothoracic region (slice thickness, 3 mm).

The MRI and ultrasound images were interpreted by two separate independent examiners, each of whom had more than 10 years of experience in their specialty.

To estimate the frequency of visualization and to allow a comparison with MRI, the brachial plexus was divided into the following segments: the foraminal zone (nerve roots); the interscalene space (from the junction of the nerve roots to the formation of trunks); the costoclavicular space, comprising the supraclavicular region (trunks and divisions) and infraclavicular region (anterior and posterior divisions, as well as the cords); and the retropectoralis minor space (cords).

**Figure 7:**
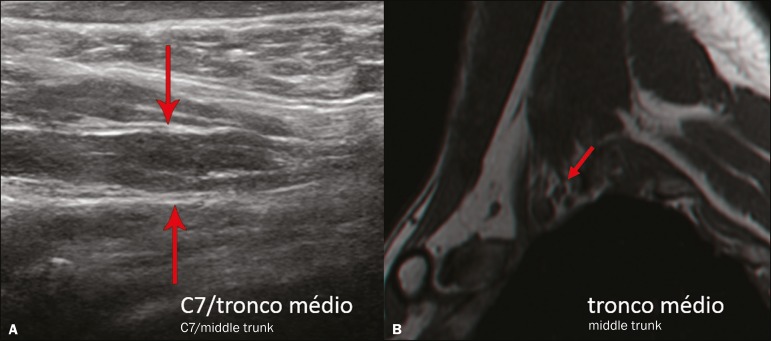
**A:** Ultrasound in the longitudinal plane showing the C7 root/middle trunk (arrows) in the interscalene space-visualization score of 2. **B:** MRI in the sagittal plane showing the middle trunk (arrow).

**Figure 8:**
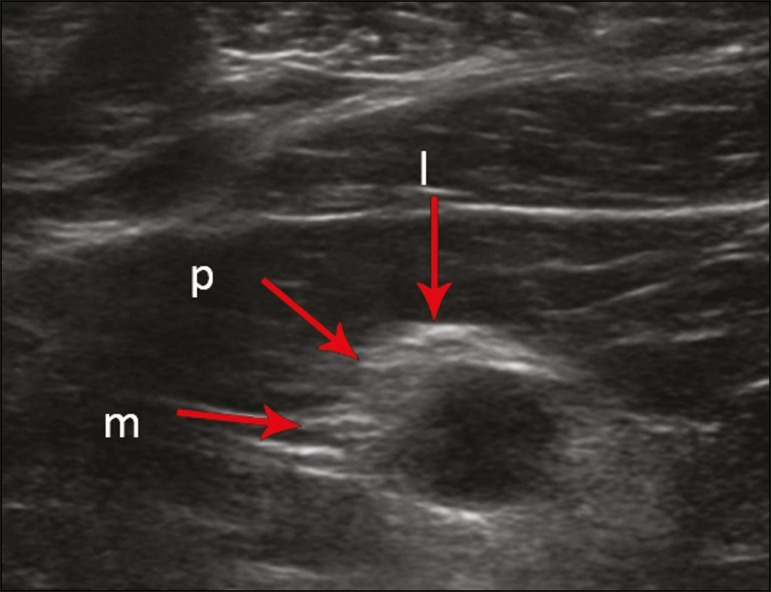
Ultrasound of the retropectoralis minor space, showing the medial (m), lateral (l), and posterior (p) cords (arrows), with a visualization score of 1.

The visualization of the structures was categorized on the basis of a scoring system (Figures 7 and 8): 0 = no segments identified; 1 = partially identified segments or undefined images; and 2 = well-visualized segments.

In the assessment of interobserver agreement-for ultrasound and MRI-we calculated the kappa statistic, which is useful for the categorization of the variability in the interpretation of two separate datasets. However, the kappa statistic can be inapplicable in some situations, such as in the presence of null categories. In those situations, we also considered the agreement assessment in isolation from the obtained data. In the comparative analysis, the kappa statistic was also used in order to determine the agreement between the more experienced ultrasound and MRI observers (observer 1, in both cases) and the less experienced ultrasound and MRI observers (observer 2, in both cases)

## RESULTS

The structures that were best visualized by ultrasound were the cervical roots and their exit passages through the intervertebral foramens to the interscalene space, together with the upper and middle trunks. Among those, the C6 and C7 roots were most easily visualized by both observers, presenting a visualization score of 2 in more than 75% of the sample. In contrast, the C5 and C8 roots presented a visualization score of 2 in up to 65% of the sample. The poorest visualization was of the T1 roots, which were not visualized in 100% of the sample by either observer. Table 1 shows the proportional distribution of the visualization score of 2 reported by the two ultrasound observers.

**Table 1:** Proportional distribution of a visualization score of 2 assigned to the brachial plexus segments by ultrasound observers 1 and 2.

Brachial plexus segment	Observer 1	Observer 2
Right C6 root	95	80
Left C6 root	90	75
Right C7 root	100	95
Left C7 root	100	95
Right upper trunk	80	60
Left upper trunk	80	80
Right middle trunk	95	95
Left middle trunk	90	95

In the ultrasound analysis of the trunks of the plexus, the middle trunk was the one that was the most well visualized, with a visualization score of 2 in 95% of the sample. The upper trunk had a mean visualization score of 2 in 70% of the sample, whereas the lower trunk had a visualization score of 2 in up to 65%. The anterior and posterior divisions of the trunks were the plexus segments with the worst visualization score, revealing that ultrasound was quite limited in the identification of these structures. Regarding the cord segment of the brachial plexus, the proportional distribution of the visualization scores was quite heterogeneous, with no predominance of a score of 0 or 2, in the costoclavicular (infraclavicular) space or in the retropectoralis minor space.

In the MRI visualization score analysis, all of the cervical roots, from C5 to T1, had a visualization score of 2 in more than 80% of the sample, from both observers, as did the upper, middle, and lower trunks, as well as the lateral, posterior, and medial cords, the most experienced observer (observer 1) registering a visualization score of 2 for all the nerve roots from C5 to T1. Table 2 shows the proportional distribution of an MRI visualization score of 2, by observer. However, for both observers, the MRI visualization score of 2 was distributed heterogeneously among the divisions of the brachial plexus. For the upper and middle trunk divisions, observer 1 registered a visualization score of 2 in up to 95% of the sample, registering that same score in 100% for the lower trunk division. In contrast, observer 2 registered a visualization score of 1 for most of the trunk divisions.

**Table 2:** Proportional distribution of a visualization score of 2 assigned to the brachial plexus segments by MRI observers 1 and 2.

Brachial plexus segment	Observer 1	Observer 2
Right and left C5 roots	100	80
Right and left C6 roots	100	100
Right and left C7 roots	100	100
Right and left C8 roots	100	100
Right and left T1 roots	100	100
Right upper trunk	85	100
Left upper trunk	85	95
Right middle trunk	100	95
Left middle trunk	100	100
Right and left lower trunks	100	100

For the ultrasound findings, the interobserver agreement was almost perfect (kappa > 0.81) or non-applicable, with perfect agreement regarding the C7 root, T1 root, middle trunk, lower trunk, and the trunk divisions (Table 3). For the MRI findings, the interobserver agreement was perfect or almost perfect (kappa > 0.81) for the cervical roots, trunks, and cords (Table 3).

**Table 3:** Level of interobserver agreement for ultrasound and MRI, almost perfect (kappa > 0.81) or not applicable, with perfect agreement for the brachial plexus segments.

Interobserver variable (for ultrasound)	Kappa statistic	Level of agreement	Agreement
Right and left C7 roots	Not applicable	-	95%
Right and left T1 roots	Not applicable	-	95%
Right middle trunk	1.000	Almost perfect	100%
Right lower trunk	0.920	Almost perfect	95%
Right upper anterior and posterior divisions; left lower anterior and posterior divisions	Not applicable	-	100%
Left upper anterior and posterior divisions; right lower anterior division	Not applicable	-	95%
Interobserver variable (for MRI)	Kappa statistic	Level of agreement	Agreement
Right and left C6, C7, C8, and T1 roots	Not applicable	-	100%
Right middle trunk	Not applicable	-	95%
Left middle trunk	Not applicable	-	100%
Right lower trunk	Not applicable	-	90%
Left lower trunk	Not applicable	-	100%
Right lateral cord (costoclavicular space)	Not applicable	-	95%
Left lateral cord (costoclavicular space)	Not applicable	-	90%
Right posterior cord (costoclavicular space)	Not applicable	-	75%
Left medial cord (retropectoralis minor space)	1.000	Almost perfect	100%
Right and left lateral cords (retropectoralis minor space)	Not applicable	-	95%
Right posterior cord (retropectoralis minor space)	Not applicable	-	95%
Left posterior cord (retropectoralis minor space)	1.000	Almost perfect	100%

In the interobserver analysis comparing only the most experienced ultrasound and MRI observers, the C6 root, C7 root, and bilateral middle trunk were equivalent with a concordance higher than 85% between the observers. As can be seen in Table 4, there was equivalence for the other brachial plexus segments as well (kappa of 0.65-0.80): the right C5 root; the bilateral upper trunk; the left lower trunk; the right upper anterior and posterior divisions; and the left lateral cord.

**Table 4:** Equivalence of interobserver agreement for the brachial plexus segments between ultrasound observer 1 and MRI observer 1.

Variable	Kappa statistic	Agreement	Result
Right C5 root	Not applicable	65%	Equivalent
Right C6 root	Not applicable	95%	Equivalent
Left C6 root	Not applicable	90%	Equivalent
Right C7 root	Not applicable	100%	Equivalent
Left C7 root	Not applicable	100%	Equivalent
Right upper trunk	Not applicable	70%	Equivalent
Left upper trunk	Not applicable	70%	Equivalent
Right middle trunk	Not applicable	95%	Equivalent
Left middle trunk	Not applicable	90%	Equivalent
Left lower trunk	Not applicable	65%	Equivalent
Right upper anterior division	Not applicable	65%	Equivalent
Right posterior superior division	Not applicable	80%	Equivalent
Left lateral cord (costoclavicular space)	Not applicable	60%	Equivalent

## DISCUSSION

On the basis of the results we obtained for the ultrasound visualization scores of the brachial plexus segments, three different groups of nerve structures can be identified: well-visualized segments (visualization score of 2); segments with intermediate visibility (visualization scores ranging from 0 to 2); and segments with unsatisfactory visibility (visualization score of 0). In the first group, the most well visualized segments were the C6 root, C7 root, and the middle trunk, all of which had a visualization score of 2 in the majority of the sample. The exception was the T1 root, which was not visualized in any of the cases in our sample. These findings are consistent with those in the literature in terms of the difficulty in clearly visualizing the exit passages of roots C8 and T1^(^^7^^-^^9^^)^. The medial, lateral and posterior cords of the plexus were in the intermediate visibility group. In those segments, the variations in the visualization score may be attributed to the greater level of experience of one of the observers. The unsatisfactory visualization group comprised the anterior and posterior divisions of the trunk, which vary in their locations, making it difficult to identify the branches individually, because of the complexity of the anatomical network^(^^1^^-^^10^^)^. Therefore, we observed that acceptable segment visualization correlated with the superficial region and caliber of the nerve structures. Likewise, roots C6 and C7 (and consequently the middle trunk) were identified with more clarity than were the anterior and posterior divisions of the trunks. For the plexus cords, factors that increase technical limitations for adequate visualization are patient biotype, proximity to vascular structures, and location in the costoclavicular or deeper regions of the retropectoralis minor space.

On the basis of the results we obtained for the MRI visualization scores of the brachial plexus segments, two different groups of nerve structures can be identified: well-visualized segments; and intermediate or poorly visualized segments. For the well-visualized segments, both observers registered a visualization score of 2 for the proximal segments of the brachial plexus, which includes the exit passages of the nerve roots from their intervertebral foramina (C5-T1), as well as the trunks and cords. Intermediate or poorly visualized segments include the anterior and posterior divisions, for which there was low interobserver agreement in terms of the visualization scores. We noted that observer 2, who was less experienced, assigned visualization scores of 1 or 2 to the anterior and posterior divisions (each score being assigned to approximately 40% of the sample), whereas the more experienced observer (observer 1) assigned a visualization score of 0 to those divisions in approximately 20% of the sample. As was true for the ultrasound findings, anatomical variations or complexities of these divisions are the main reasons for the unsatisfactory visualization scores.

To our knowledge, there have been no studies aimed specifically at evaluating the degree of visualization of the divisions, either on MRI or on ultrasound. Our study shows that visualization of these segments is unsatisfactory on ultrasound and MRI. Our results also underscore the fact that, although the sensitivity of ultrasound for the visualization of some segments of the plexus can be comparable to that of MRI, the latter provides better visualization of most of the structures that compose the brachial plexus.

The main aspects that should be considered in the analysis of any diagnostic method are its efficacy and its limitations. Although MRI is currently the reference method for the study of the brachial plexus, it is costly and relatively time-consuming if all the segments of the brachial plexus are to be evaluated, not to mention the well-known contraindications to its use^(^^10^^)^. As an alternative to MRI, ultrasound can be used in specific cases in which the objective is to evaluate the proximal segment of the brachial plexus, in order to detect impairments along its path in the cervical region, especially those in which the patient is clinically limited and cannot undergo MRI, as well as those in which the results of the neurophysiological assessment are inconclusive^(^^11^^)^, as they often are in neonates with plexus palsy. Ultrasound can also be useful to guide procedures involving nerve block anesthesia, because it allows the nerve roots and their anatomic variations to be identified, thus preventing complications such as vascular perforation^(^^12^^-^^14^^)^.

There is a need for greater dissemination of knowledge related to ultrasound and more extensive training of radiologists in its use in the visualization of the brachial plexus. Such advances could make ultrasound a method that is used more routinely in the evaluation of the brachial plexus, given the specific criteria that justify its use as a complementary tool for clinicians and surgeons who treat patients with brachial plexus injuries.

## CONCLUSION

The results of our analysis of the visualization of the brachial plexus on ultrasound allowed us to conclude that accuracy of ultrasound was high in the proximal segments, which comprise the ventral branches of the C5, C6, and C7 nerve roots, as well as the upper and middle trunks in the lateral region of the neck.

Ultrasound demonstrated a high degree of concordance with MRI in the cervical ventral branches of the C6 and C7 nerve roots, as well as in the middle bilateral trunk. For the visualization of the other brachial plexus segments, there was no high level of agreement among the observers, although the visualization scores were always lower for the ultrasound observer.
